# Linking the ECRIN Metadata Repository with the BBMRI-ERIC Directory to connect clinical studies with related biobanks and collections

**DOI:** 10.12688/openreseurope.17131.2

**Published:** 2024-10-07

**Authors:** Christian Ohmann, Steve Canham, Kurt Majcen, Vittorio Meloni, Luca Pireddu, Alessandro Sulis, Giovanni Delussu, Francesca Frexia, Petr Holub

**Affiliations:** 1 ECRIN, European Clinical Research Infrastructure Network, Paris, 30 Bd. Saint Jacques, 75014, France; 2 Biobanking and Biomolecular Resources Research Infrastructure – European Research Infrastructure Consortium, Graz, 8010, Austria; 3 Center for Advanced Studies Research and Development in Sardinia, Pula, Sardegna, Italy

**Keywords:** clinical study, clinical trial, biobank, collection, metadata, linkage, BBMRI-ERIC, ECRIN

## Abstract

**Background:**

There is much value to be gained by linking clinical studies and (biosample-) collections that have been generated in the context of a clinical study. However, the linking problem is hard because usually no direct references between a clinical study and an associated collection are available.

**Methods:**

The BBMRI-ERIC Directory and the ECRIN Metadata Repository (MDR), already include much of the information required to link clinical studies and related sample collections. In this study, we present the work performed to find and implement those links across existing corresponding records in the two systems. The linking process between MDR studies and related collections in the BBMRI-ERIC Directory started with exploring linkage in both directions – searching the BBMRI-ERIC Directory for candidate hits to try to link with MDR records, and searching the ECRIN MDR for candidate hits to try to link with Directory collections. Thereafter, a systematic search through the BBMRI-ERIC Directory was performed.

**Results:**

The investigation of linkages in both directions resulted in a limited but promising number of linkages. The results of the systematic search of the Directory identified linkage of 202 studies, spanning 284 collections.

**Conclusions:**

The analysis with existing data sources indicated that links between the BBMRI-ERIC and ECRIN collections exist, but also that they would be difficult to continuously identify and maintain without a great deal of manual work which neither organisation could support. The question arises whether, in the future, systems could be put into place to make the exchange of information and the linkage of identifiers almost automatic.

## Introduction

The Biobanking and Biomolecular Resources Research Infrastructure – European Research Infrastructure Consortium ( BBMRI-ERIC) Directory collects information about biobanks throughout Europe as well as the biosample collections and data those biobanks make available for research (currently, over 400 biobanks are registered, collectively making available over 1400 sample collections) ^1^. The data model of the BBMRI-ERIC Directory is currently based on the Minimum Information About BIobank data Sharing ( MIABIS) Core 2.0 metadata schema and is focused on describing two principal entities: biobanks (institutions) and collections (within biobanks containing data/samples) ^2^.

The European Clinical Research Infrastructure Network ( ECRIN) Metadata Repository – MDR – collects metadata about clinical research studies and digital objects related to them ^3^. The MDR is based upon the ECRIN metadata schema. It contains metadata from 800,000 clinical research studies (around 80% interventional clinical trials and 20% observational studies) and 1.3 million related digital objects, such as trial registry entries, journal papers, results summaries, protocols etc. The MDR covers all primary World Health Organisation (WHO) registries, ClinicalTrials.gov, Pubmed and two repositories (Yale University Open Data Access (YODA), Biologic Specimen and Data Repository Information Coordinating Center (BioLINCC)).

There is much value to be gained by enhancing the metadata in both these systems to allow linking of entities between BBMRI-ERIC and ECRIN in those cases where samples have been generated in the context of a registered clinical study. This linking would allow users of the MDR to easily identify studies that had associated samples, listed in the BBMRI-ERIC Directory, and users of the BBMRI-ERIC Directory to find information more easily about the studies that generated samples in collections of interest.

The benefit of linking clinical studies/clinical trials to biobanks and sample collections is outlined in the following user story. A clinical trial was performed to investigate whether an interactive internet-based intervention strategy, targeting vascular and lifestyle-related risk factors, can lead to improvement of cardiovascular risk profiles and prevention of cardiovascular diseases, and whether this in turn may prevent or delay the onset of cognitive decline and dementia ^4^. This trial entitled “Healthy aging through internet counseling in the elderly” *(sic)* has been registered in a trial registry ( ISRCTN48151589). After identifying this trial in the ECRIN metadata repository (MDR), several digital objects are presented by the system that are directly linked to this trial: the Statistical Analysis Plan, the Patient Information sheet, website and journal articles (n=6). In the trial registry and MDR there is no mention, however, of the biosamples collected in the context of this study. If a researcher is interested in such material and thus wants to identify and access a biobank collection, he/she must find it through other means; for instance, the researcher can search the BBMRI-ERIC Directory for collections linked to this study ( bbmri-eric:ID:NL_AMCBB:collection:AB16-005) – making the best of the available metadata in the hopes of being successful. Currently, there is no direct and specific link between a clinical trial in the MDR and a biobank/collection in the BBMRI-ERIC Directory, so this search can be time-consuming and even difficult due to lack of metadata interoperability across the two repositories, potentially yielding different representations of the same entities or even simply different spellings, naming conventions etc. Likewise, if a researcher has identified a collection in the BBMRI-ERIC Directory and would like to find more specific information on the underlying trial, he/she must try to identify the trial through a manual search of the MDR, or the International Clinical Trials Registry Platform (ICTRP) or some other resource (e.g., PubMed), coping with difficulties due to the lack of standardisation across the various types of registries.

The situation would be simplified tremendously and become machine-actionable if the clinical trial and the related biobank collection were directly linked. This link can be established by adding the biobank collection ID to the MDR, and conversely adding the study identifiers in the MDR to the biobank collection in the BBMRI-ERIC Directory. Then, a researcher interested in a biobank collection found in the BBMRI-ERIC Directory would be able to also access the clinical trial directly in the MDR, and thus also access various digital objects and documents related to the trial – for example, the patient information sheet and a specific journal article. Conversely, a researcher that has identified a trial in the ECRIN MDR and wants to know whether any biobank collections are available will automatically have access to this information as well as a direct link to the collection’s page in the BBMRI-ERIC Directory.

Both systems, the BBMRI-ERIC Directory and the ECRIN MDR, already include much of the information required to link clinical studies and sample collections. In this study, we present the work performed to find and implement links across existing corresponding records in the two systems, charting a path to simplify the activity of the researcher in the previously described use case. At this stage, we have limited the work to COVID-related studies and collections. In the following sections, we describe the difficulties posed by the problem of identifying corresponding records and the procedure implemented to mine the two repositories in search for them; this process consisted in a preliminary analysis, which was performed to investigate the feasibility of a more extensive linking between the two systems, followed by an extensive systematic search for all corresponding COVID-19 related studies and biosample collections. We present the outcomes of the linking process applied to the MDR and Directory and, finally, we discuss implications and future prospects for these initial results.

## Methods

The linking problem was considered as hard because usually no direct references between a clinical trial/clinical study in the MDR and an associated collection in the BBMRI-Directory are available. To cope with this, the linking process between MDR studies and related collections in the BBMRI-ERIC Directory was performed in two steps:

- Exploring linkage (in each direction)
- Systematic search through BBMRI-ERIC Directory

### Exploring linkage (in each direction)

This process can be characterised as explanatory. The aim of the first step in our process was to determine whether direct links between collections in the BBMRI-ERIC Directory and registered clinical trials would be possible, and in how many cases. For this purpose, we implemented a linking process in two directions – searching the BBMRI-ERIC Directory on some key terms for candidate hits to try to link with MDR records, and searching the ECRIN MDR focussing on COVID-19 studies (to reduce workload) registered in a European Registry to see if they could be found in the BBMRI-ERIC Directory.. A summary of the process is given in Table 1:

**Table 1.** Summary of the process.

Phase	Description	Process
1	Exploring linkage (in each direction)	- Scan of the BBMRI-ERIC Directory for specific keywords related to clinical studies - Match of a sample to clinical studies in the MDR (using MDR portal search mechanisms)
		- Scan of the MDR for COVID-19 clinical studies registered in a European registry - Match to collections in the BBMRI-ERIC Directory (via a search library for the Directory, largely using study acronyms)
2	Systematic search of BBMRI-ERIC Directory	- Systematically searching descriptions and keywords in the BBMRI-ERIC Directory and looking for an unambiguous match to clinical studies in the MDR

### Exploring linkage from BBMRI-ERIC Directory to ECRIN MDR

For searching biobanks linking to studies on BBMRI-ECRIN side, we used the full-text searching capabilities of the Directory catalogue to search for a set of purpose-designed query strings in any data/sample collections. The full-text search script targeted all text fields of the catalogue; the code is available on GitHub ( https://github.com/BBMRI-ERIC/directory-scripts).

The resources identified in the BBMRI-ERIC Directory with the search pattern “clinical trial” were investigated in detail for possible links to clinical studies in the MDR. The free text pertaining to the record was assessed to explore whether a linked clinical trial could be identified. Main and specific keywords of the resource were entered into the ECRIN MDR search system. The descriptions of the clinical trials identified by the MDR were compared manually with the descriptions in the BBMRI-ERIC Directory. If in reasonable time (no more than 10 minutes), a unique link between the collection in the BBMRI-ERIC Directory and a specific clinical trial in the MDR could be made, the URL of the clinical trial registration was mapped to the collection. The threshold of 10 minutes was used for practical reasons, admitting that this is not the best strategy due to variable length of descriptions in ECRIN MDR and BBMRI-ERIC Directory.

### Exploring linkage from ECRIN MDR to BBMRI-ERIC Directory

For searching studies on ECRIN side that link to biobanks/collections, we started with a search for candidate studies in the MDR, to then determine whether a linked biosample collection could be identified in the Directory. We began by identifying those studies in the MDR that had some reference to COVID-19, coronavirus, or SARS-2 in their titles or listed topics (n=15,763). 134 of these were removed because they had start dates prior to 2020 (and so were pre-pandemic), unless they explicitly mentioned COVID-19 in the title (in which case they are presumed to have been studies adapted to include COVID-19, or which had a wrong date entered accidentally). Identifying the distinct studies in this dataset (because some studies are entered into more than one trial registry, and thus may have multiple records) yielded a final global COVID set of 15,201. From these studies, a subset of 3,893 were selected by the presence of an acronym – because it was suspected (and later confirmed) that it would be more accurate to try and match acronyms than fragments of full titles. Out of that group, 630 were identified as being registered in a European, or largely European trial registry: i.e. the EU Clinical Trials Register (EU CTR), ISRCTN (International Standard Randomised Controlled Trial Number), German Clinical Trials Register (DRKS), and Dutch Trial Register. This group was seen as particularly relevant to BBMRI-ERIC, which mainly catalogues biobanks and collections within its member states, which are to a large extent located in Europe.

The search for linkable studies in the MDR provided a list with potentially identifying information to be used to search the BBMRI-ERIC Directory, or at least provided information that could be further processed before checking it against the Directory. The information consisted of study records with the attributes of (internal) MDR-ID, Registry ID or IDs, acronyms and the long title used for the trials. The attributes of the list of candidates from MDR was then used to scan a download of the full BBMRI-ERIC Directory metadata. The scan was done once with exact matching and then again with truncation or embedding within certain characters like e.g. “- , ; . ' :”.

### Systematic search of BBMRI-ERIC Directory

Once the preliminary analysis confirmed the linkage potential between the two metadata collections, we proceeded with the second, more thorough step in our process to find and link corresponding clinical studies and biospecimen collections. We performed a systematic search of the public BBMRI-ERIC Directory (i.e. all biobanks and collections). Using the collection name, and the description, it was relatively straightforward to identify collections that, at least potentially, were linked to clinical research studies (rather than, for instance, population- or diagnosis-based sample collections). In many cases, the title of the collection included a named study or related to a comparison of different treatments or cohort groups.

Our investigation demonstrates the difficulty in unambiguously linking registered sample collections to specific registered studies. To achieve our cross-linking goal while avoiding potential mismatch errors, we defined and executed the following procedure.

- If a description in the Directory included a direct reference to a trial registry ID (a few do), that information was used to identify the study directly.
- If a description in the Directory included a link to a web site or a journal paper (again some do), that resource could often be used to retrieve a trial registry id.
- Otherwise, an attempt was made to match key words in the BBMRI-ERIC Directory collection title or description with words in MDR titles. This was done manually using direct interrogation of the MDR database. In some cases, the BBMRI-ERIC description is the same or almost the same as the ‘full’ study title in the MDR (the scientific or protocol title). In other cases, the BBMRI-ERIC title was a study acronym, which very often needed to be disambiguated through further work. Note that the default MDR title for a study is the usually shorter ‘public title’, and so may not always obviously match the BBMRI-ERIC collection title.
- Disambiguation of acronyms was also attempted by using the detailed description in the BBMRI-ERIC Directory, when available, and matching that to the study’s scientific title or description.
- In some cases, disambiguation was also achieved by matching the sponsor of a study to the host organisation for the repository.

## Results

### Exploring linkage from BBMRI-ERIC Directory to ECRIN MDR

The search terms used (applied with both left and right truncation) and then number of corresponding results returned from the BBMRI-ERIC Directory are summarised in Table 2 below. The scan of the BBMRI-ERIC Directory was performed by KM with support from a colleague. The technical preparation and data check of the search on the BBMRI side needed around two days.

**Table 2.** Search patterns used to scan the BBMRI-ERIC Directory in the exploring linkage and corresponding number of search hits. (Search patterns were left and right truncated.)

No.	Search pattern	No. of resources found
1	*random*	7
2	*blind*	30
3	*clinical trial*	135
4	*clinical stud*	0
5	*compar*	84
6	*investig*	126
-	Total	382

### Exploring linkage from ECRIN MDR to BBMRI-ERIC Directory

For each attribute, we report the number of selected MDR records for which we found a text-based match in the BBMRI-ERIC Directory metadata. The results of this process are shown in Table 3.

**Table 3.** Results from preliminary search process starting from MDR candidates.

Attribute name	Number of search hits from Directory collection metadata
Registry-ID	0
Acronym	2530 (This included lots of false positives, as anticipated, due to use of commonly used, rather general terms, like ‘achieve’, ‘act’, ‘COVID’, ‘trust’ etc.; also acronyms are often not created in an unambiguous way)
Title	2 (Small number expected as searching with rather long phrases including many words)

The resources identified in the BBMRI-ERIC Directory were compared with resources in the MDR to identify possible matches between clinical studies/clinical trials and biobanks/collections. Only 7 studies were identified unambiguously ^5^.

### Systematic search of BBMRI-ERIC Directory

The results of the systematic search of Directory, after some de-duplication and removal of what appeared to be spurious ‘placeholder’ collection entries, was the identification of 202 studies, spanning 284 collections ^5^. The systematic search of the BBMRI-ERIC Directory entries against the MDR was performed by SC. It took about 2 days. The linkage data (along with related information such as collection and biobank IDs, MDR IDs, and sample details) have meanwhile been imported into ad hoc prototypes of both the MDR (search by linked data object = “available samples”) and a functionally extended version of BBMRI-ERIC Directory to showcase the links. At the time of publication this prototype version of Directory is accessible at the URL https://rd-demo.directory.bbmri-eric.eu/; note however that the authors are in the process of integrating the new functionality in the production instance of Directory, after which the prototype will be dismissed.

## Discussion

The FAIR guiding principles for scientific data management and stewardship state in its section on interoperability that data needs to be integrated with other data (I3 from 6). The linking across identifiers has been investigated in different projects (e.g., THOR, FREYA) and conceptual models have been developed ^7, 8^. The different approaches to how data should be interlinked are described in detail in the FAIR cookbook ^9^. In the ECRIN-BBMRI use case, mapping is performed between databases containing different information about the same entity, here a clinical trial/clinical study. In the MDR a clinical trial/clinical study is identified by a study identifier (sometimes there are several study identifiers) and in the BBMRI-ERIC Directory usually indirectly, e.g. by mentioning in free text in the description or other metadata fields or sometimes by direct reference to a clinical trial/clinical identifier. Consequently, direct mapping was not possible in most cases and considerable manual input was needed.

The results of this exercise show that quite a substantial number of collections in the BBMRI-ERIC Directory were created in the context of specific clinical studies. Considerable manual input was needed, however, to identify this linkage. The retrospective approach taken in this study is, therefore, only ever likely to be useful as a demonstration of the potential usefulness of linking the two systems. To enable a more realistic and complete linkage system, it will be necessary to include data points that support linkage (i.e. a clinical trial identifier) within the metadata of the biobank collections, at the time when metadata are created in the process of onboarding to the BBMRI-ERIC Directory. It was estimated that there are approximately another 100 studies/trials in the MDR for which corresponding studies exist in the Directory, but which could not be unambiguously linked because of insufficient information. The full extent of the overlap of the two systems is therefore – currently – estimated at about 300 studies with more than 300 collections involved. Doing the aforementioned matching also took several days. So, given the time required, this is clearly a one-off exercise that needs to be replaced by an (semi-) automatic process as soon as possible.. Whether AI or ML techniques could help needs to be sorted out.

Approaches using ‘fuzzy matching’ may be worth exploring in this context. The major task of the process for the systematic search, was disambiguation. Study acronyms and ‘key’ words or phrases in titles were often far from unique in the MDR data, so it was necessary to investigate further, as described. In those circumstances fuzzy matching could increase the workload, by identifying an increased number of ‘false positives’. Machine learning approaches to the disambiguation process could also be investigated, though the number of links identified (about 200) may not form a large enough training data set for this to be viable, especially given that the text in both the BBMRI and MDR descriptions is highly variable in length and sometimes very short.

The fundamental problem is that the source data used for each system does not routinely or consistently contain data about the entities described by the other system: the trial registry data used by the MDR only rarely refers to sample collections, while the data about samples in the BBMRI-ERIC Directory, even if those samples were gathered in the context of a clinical research study, rarely identify the study clearly.

For automatic linkage to take place, one or both systems needs to include the identifiers used by the other system, or at least something that can be mapped to them. If that was the case the mechanics of the linkage process would be relatively straightforward, and could involve periodic interrogation of one system by the other, or making the relevant data available, e.g. via an API. Automatic linkage between the systems is therefore blocked by the absence of the relevant metadata, not by a technical system issue.

In practical terms it would be difficult for the MDR data to contain consistent references to samples. The MDR has over 20 source systems, and has no control over the metadata those sources provide – it can only collect what is available. Trial registry data must comply with WHO requirements, but these do not include the need to list available samples. Even if they did, there is no consistent system, globally, for describing samples and their availability, and there seems little prospect of one being developed and mandated by the WHO.

Including unique trial Ids in the BBMRI source data, when relevant, would seem more possible, though still difficult. Trial registry Ids do provide unique public identifiers for clinical research studies. (Not all the studies listed in the BBMRI-ERIC Directory would be registered, but if they were not they would not be included in the MDR, so no linkage would be possible in any case.) The BBMRI dataset is much smaller (about 1700sample collections versus 800,000+ study records in the MDR) and only a subset of these is linked to a defined study, as opposed to being a population or disease based collection. Educating and persuading source biobanks to include relevant trial Ids in returned data would not be easy, and would require resources, but it does at least appear feasible. Linkage between the two systems in the future would seem dependent on such a development.

Attempting to link the data of the ECRIN MDR and BBMRI-ERIC’s Directory has underlined the need for metadata schemas that can support data sharing between systems, and the consequent need – even between closely related disciplines such as clinical research and biobanking – for such schemas to be shared, compared and discussed.

Finally, GDPR is not an issue for this exercise. The linked data is already in the public domain, in either the MDR or the BBMRI directory. For the MDR at least the source data is also public. The data includes the names of individuals, largely as investigators or contacts, but this is not ‘sensitive’ data – just recognition of professional roles that are public in any case. As such GDPR has little or no impact.

## Data Availability

Zenodo: EOSC-Future Test Science Project "META-COVID": Linked resources between BBMRI-ERIC Directory and ECRIN Metadata Repository, https://doi.org/10.5281/zenodo.10435560 ^5^. Data are available under the terms of the Creative Commons Attribution 4.0 International license (CC-BY 4.0). **Source code available from:** https://github.com/BBMRI-ERIC/directory-scripts **Archived source code at time of publication:** https://doi.org/10.5281/zenodo.10610005 ^10^. **License:** LGPL – 3.0
