# Supplementary material for: Linking the ECRIN Metadata Repository with the BBMRI-ERIC Directory to connect clinical studies with related biobanks and collections
Source: Open Res Eur. 2024 Oct 7;4:50. Originally published 2024 Mar 4. [Version 2] doi: 10.12688/openreseurope.17131.2 (PMC11467644; doi:10.12688/openreseurope.17131.2)
Supplement: Peer review history

# Reviewer report: Reviewer response for version 2

**DOI:** 10.21956/openreseurope.20086.r44949

**Published:** 2024-10-17

**Authors:** Catherine McCarty

**Affiliations:** 1 University of Minnesota Medical School, Duluth, USA

The authors have adequately addressed reviewer comments.

Is the study design appropriate and does the work have academic merit?

Yes

Is the work clearly and accurately presented and does it cite the current literature?

Yes

If applicable, is the statistical analysis and its interpretation appropriate?

Not applicable

Are all the source data underlying the results available to ensure full reproducibility?

Yes

Are the conclusions drawn adequately supported by the results?

Yes

Are sufficient details of methods and analysis provided to allow replication by others?

Partly

Reviewer Expertise:

epidemiology, health services research

I confirm that I have read this submission and believe that I have an appropriate level of expertise to confirm that it is of an acceptable scientific standard.

# Reviewer report: Reviewer response for version 2

**DOI:** 10.21956/openreseurope.20086.r44950

**Published:** 2024-10-10

**Authors:** Maryam Abdollahyan

**Affiliations:** 1 Queen Mary University of London, London, UK

The authors have addressed my concerns. Many thanks.

Is the study design appropriate and does the work have academic merit?

Yes

Is the work clearly and accurately presented and does it cite the current literature?

Partly

If applicable, is the statistical analysis and its interpretation appropriate?

Not applicable

Are all the source data underlying the results available to ensure full reproducibility?

Yes

Are the conclusions drawn adequately supported by the results?

Yes

Are sufficient details of methods and analysis provided to allow replication by others?

Yes

Reviewer Expertise:

Health Informatics, Biobank Infrastructure

I confirm that I have read this submission and believe that I have an appropriate level of expertise to confirm that it is of an acceptable scientific standard.

# Reviewer report: Reviewer response for version 1

**DOI:** 10.21956/openreseurope.18517.r39792

**Published:** 2024-05-25

**Authors:** Valentina Colcelli

**Affiliations:** 1 National Research Council of Italy, Florence Research Area, Italy

The article is interesting and really well written because it is clear in its objectives and methodology, but in my opinion, it should take GDPR into consideration. That is to say, some thought should be given to ensuring that the data protection guarantee standards are ensured during the actual implementation of the link between the two infrastructures. At this moment, no part of the paper takes into consideration this guarantee.

Is the study design appropriate and does the work have academic merit?

Yes

Is the work clearly and accurately presented and does it cite the current literature?

Yes

If applicable, is the statistical analysis and its interpretation appropriate?

Not applicable

Are all the source data underlying the results available to ensure full reproducibility?

No source data required

Are the conclusions drawn adequately supported by the results?

Yes

Are sufficient details of methods and analysis provided to allow replication by others?

Partly

Reviewer Expertise:

Juridical and ethical skills

I confirm that I have read this submission and believe that I have an appropriate level of expertise to confirm that it is of an acceptable scientific standard, however I have significant reservations, as outlined above.

# Response

**Published:** 2024-10-01

**Authors:** Christian Ohmann

**Affiliations:** European Clinical Research Infrastructure Network, Paris, France

A section referring to the GDPR was added to the discussion.

# Reviewer report: Reviewer response for version 1

**DOI:** 10.21956/openreseurope.18517.r39798

**Published:** 2024-05-25

**Authors:** Catherine McCarty

**Affiliations:** 1 University of Minnesota Medical School, Duluth, USA

This is an interesting exercise with implications for funders and researchers.  The authors attempted to link entries in the BBMRI-ERIC directory and the ECRIN Metadata Repository (MDR) through a two-stage process. Linking information from clinical studies with biological samples would provide a powerful tool to for future research.  In stage 1, they searched the BBMRI-ERIC directory using full-text searching capabilities.  The free text of resources in the directory with the search pattern "clinical trial" were then investigated to identify a linked clinical trial. Main and specific keywords from the resources were then entered into the ECRIN MDR search system. Descriptions of clinical trials were then manually compared between between MDR and BBMRI-ERIC. 

 The authors do not mention if the manual process was completed by one person or multiple individuals, and whether inter- or intra-rater reliability was performed.  It would also be helpful to know how long the manual process took. This is important in the discussion for considering sustainability.

Is the study design appropriate and does the work have academic merit?

Yes

Is the work clearly and accurately presented and does it cite the current literature?

Yes

If applicable, is the statistical analysis and its interpretation appropriate?

Not applicable

Are all the source data underlying the results available to ensure full reproducibility?

Yes

Are the conclusions drawn adequately supported by the results?

Yes

Are sufficient details of methods and analysis provided to allow replication by others?

Partly

Reviewer Expertise:

epidemiology, health services research

I confirm that I have read this submission and believe that I have an appropriate level of expertise to confirm that it is of an acceptable scientific standard, however I have significant reservations, as outlined above.

# Response

**Published:** 2024-10-01

**Authors:** Christian Ohmann

**Affiliations:** European Clinical Research Infrastructure Network, Paris, France

The persons involved in the manual process were included in the text. The approximate time effort to do the manual search was added to the text.

# Reviewer report: Reviewer response for version 1

**DOI:** 10.21956/openreseurope.18517.r39793

**Published:** 2024-05-10

**Authors:** Maryam Abdollahyan

**Affiliations:** 1 Queen Mary University of London, London, UK

The research article describes a manual approach for linkage of the ECRIN Metadata Repository (MDR) records to the BBMRI-ERIC Directory records, which facilitates the search for clinical studies and associated data and biospecimens. The authors report promising results, with over 200 studies and data/biospecimen collections matched. Furthermore, the article highlights the importance of making research outputs discoverable and the need for automating the exchange of information between such repositories in the future.

 Major comments:

- During the ‘two-stage linking process’, the search terms used to scan the BBMRI-ERIC Directory are generic (e.g., clinical trial), while the keywords used to search the ECRIN MDR are specific (e.g., SARS-2). Please comment on why, for instance, the term ‘coronavirus’ was not used to scan the BBMRI-ERIC Directory.
- In the Methods section, use of a different threshold (e.g., exact matches only or top 10 search results) instead of “reasonable time (no more than 10 minutes)” is suggested as the ECRIN MDR and BBMRI-ERIC descriptions are of variable length, and therefore, it is not clear how many descriptions were compared.
- In the Results section, the process described in the second paragraph (“The search for linkable studies…”) would fit better in the Methods section. Similarly, the number of hits reported in the Methods section (Table 1), would fit better in the Results section.
- Addition of a flow diagram that summarizes the ‘two-stage linking process’ and ‘systematic search of BBMRI-ERIC Directory’ approaches is recommended.
- Similar to the prototype ECRIN MDS, please include the URL for the extended version of the BBMRI-ERIC Directory.
- Please comment on the advantages and limitations of each approach, and how they can inform future linkage efforts and repository design (e.g., tools such as BBMRI-ERIC Locator), i.e., the last paragraph in the Discussion section can be expanded.

Minor comments:

- Some manual steps can be automated. For example, during the ‘systematic search of BBMRI-ERIC Directory’, fuzzy hashing can potentially be used to compare the BBMRI-ERIC Directory titles with the ECRIN MDR titles (step 3).
- Few grammatical errors; proofreading is recommended.

Is the study design appropriate and does the work have academic merit?

Yes

Is the work clearly and accurately presented and does it cite the current literature?

Partly

If applicable, is the statistical analysis and its interpretation appropriate?

Not applicable

Are all the source data underlying the results available to ensure full reproducibility?

Yes

Are the conclusions drawn adequately supported by the results?

Yes

Are sufficient details of methods and analysis provided to allow replication by others?

Yes

Reviewer Expertise:

Health Informatics, Biobank Infrastructure

I confirm that I have read this submission and believe that I have an appropriate level of expertise to confirm that it is of an acceptable scientific standard, however I have significant reservations, as outlined above.

# Response

**Published:** 2024-10-01

**Authors:** Christian Ohmann

**Affiliations:** European Clinical Research Infrastructure Network, Paris, France

Our approach simply tried to find likely studies within the BBMRI data by searching on some key terms. Linkage was then attempted with those that included the term ‘clinical trial’. The other focused on Covid-19 studies (because we had to restrict to a manageable number) that had been registered in a European registry, to see if they could be found in the BBMRI system. The criterion of 10 minutes was applied for practical reasons. A statement about possible bias was added. The recommendation of the reviewer was followed, and the named sections were transferred. A figure was not included but a table summarising the search strategies. The URL for the extended version of BBMRI-ERIC has been provided. The discussion was expanded according to the suggestions. The idea of fuzzy matching was included in the discussion. Proofreading was performed.
